# The Early Results of the Laparoscopic Mini-Gastric Bypass/One Anastomosis Gastric Bypass on Patients with Different Body Mass Index

**DOI:** 10.1155/2020/7572153

**Published:** 2020-03-12

**Authors:** Mohsen Mahmoudieh, Behrouz Keleidari, Naser Afshin, Masoud Sayadi Shahraki, Shahab Shahabi Shahmiri, Erfan Sheikhbahaei, Hamid Melali

**Affiliations:** ^1^Department of Surgery, Laparoscopic Surgery Fellowship, Isfahan Minimally Invasive Surgery and Obesity Research Center, Alzahra University Hospital, Isfahan University of Medical Sciences, Isfahan, Iran; ^2^Department of Surgery, Laparoscopic Surgery Fellowship, Isfahan Minimally Invasive Surgery and Obesity Research Center, Kashani University Hospital, Isfahan University of Medical Sciences, Isfahan, Iran; ^3^Student Research Committee, School of Medicine, Isfahan University of Medical Sciences, Isfahan, Iran; ^4^Department of Surgery, Laparoscopic Surgery Fellowship, Isfahan Minimally Invasive Surgery and Obesity Research Center, Amin University Hospital, Isfahan University of Medical Sciences, Isfahan, Iran

## Abstract

*Introduction*. Obesity is among the newest health matters that human beings are struggling with. Length of bypassed intestine is important in achievement of most weight loss and least nutritional and absorptive disorders. This study has aimed to assess short-term metabolic and nutritional effects of laparoscopic mini-gastric bypass/one anastomosis gastric bypass (MGB/OAGB) with a loop bypass length of 180 centimeters (cm) and compare these factors among patients with a body mass index (BMI) of 40–45 and 45–50 kilograms per square meter (kg/m^2^).

**Methods:**

25 patients were put in group 1 (BMI = 40–45 kg/m^2^) and 25 patients in group 2 (BMI = 45–50 kg/m^2^). Patients' BMI, postoperative weight, excess weight loss, and laboratory tests including fasting blood sugar (FBS), lipid profile, serum iron (Fe), ferritin, total iron-binding capacity (TIBC), 25-OH vitamin D, vitamin B12, liver function tests, and albumin were recorded preoperatively and within 3- and 6-month follow-up.

**Results:**

Weight loss and BMI reduction was significantly more in patients with higher BMI level (*P*=0.007), and excess weight loss was higher in patients with lower preoperative BMI level (*P*=0.007), and excess weight loss was higher in patients with lower preoperative BMI level (*P*=0.007), and excess weight loss was higher in patients with lower preoperative BMI level (

**Conclusion:**

Based on this study, 180-cm intestinal bypassed length works for patients with a BMI level of 40–45 and 45–50 kg/m^2^, according to their significant decrease in weight, BMI, and improving glycolipid profile.

## 1. Introduction

Obesity is among the newest health matters that human beings are struggling with today. This condition is increasingly developing in both developed and developing countries. Urbanization, sedentary lifestyle, and dietary changes are factors that have led to a growing rate of obesity [1].

Obesity has a variety of complications causing lower life expectancy including metabolic syndrome, hypertension, insulin resistance, type-2 diabetes mellitus, cardiovascular diseases, osteoarthritis, low back pain, and increased risk of malignancy. In addition, obese people are struggling with decreased self-esteem as they are not satisfied of their appearance leading to depression and reluctance of participation in social activities [2–5].

Bariatric surgery is the most effective treatment for severe obesity. This surgical procedure causes persistent weight loss, modulates complications of obesity, and increases quality of life and eventually patients' life expectancy [6].

Variety of bariatric surgeries are being performed now. Perhaps, the most popular one is Roux-en-Y gastric bypass (RYGB). Recently, it has been confirmed that the laparoscopic mini-gastric bypass/one anastomosis gastric bypass (MGB/OAGB) as an easier technique of gastric bypass is even more effective than the classic technique, RYGB [7, 8]. The RYGB has two limbs: alimentary or Roux limb and biliopancreatic limb. The MGB/OAGB has only one gastrojejunal anastomosis, which is called the biliopancreatic limb as well.

Previous studies in the RYGB field have presented that increased length of the biliopancreatic arm instead of the alimentary limb can lead to more weight reduction [9]. On the contrary, studies have shown that the total small intestine length is different in patients and biliopancreatic limb length is important to avoid complications such as malabsorption or malnutrition and to achieve more weight loss postoperatively [8–10]. In this term, different studies have assessed various lengths of the biliopancreatic arm based on small intestine length or patients' body mass index (BMI). Although studies have presented valuable results, their presentations are controversial and researchers have not declared unanimous results [11–14].

Based on the importance of selecting the best technique of gastric bypass surgery, preventing secondary malabsorption and metabolic complications, and due to controversial findings of previous studies, we aimed to assess short-term metabolic and nutritional effect of MGB/OAGB with 180-centimeter (cm) intestinal bypass length in patients with a BMI of 40–45 and 45–50 kg/m^2^.

## 2. Methods and Materials

This is a prospective cross-sectional study based on 50 patients admitted to our university hospital for MGB/OAGB in 2016–18. Approximately, 300 total bariatric surgeries are performed in our center every year, from which, 50% of them are MGB/OAGB surgery.

Inclusion criteria were age between 25 and 65 years with BMI ≥40 kg/m^2^ without any obesity-related comorbidities (e.g., diabetes mellitus, hypertension, and obstructive sleep apnea), consent declaration for participation in this study, patients' ability to tolerate laparoscopic surgery, and lack of laparotomy history.

Exclusion criteria were previous history of any bariatric surgery (e.g., gastric banding, balloon gastroplasty, or other methods), patients' unwillingness or decision change not to participate in the study, having any positive medical history for gastroesophageal reflux disease, diabetes mellitus, hypertension, thyroid, respiratory, kidney, and liver failure, and failure to follow recommended diet supplementation after MGB/OAGB.

Consent forms for participating and all-needed information about the study were given to patients prior to the surgery. This study was conducted after receiving its approval from medical ethics in the research department of our university (registration number: IR.MUI.MED.REC.1398.131).

From our extensive referral for bariatric surgery and after reviewing inclusion and exclusion criteria for all of the patients, we included 50 patients for this research. All patients were allocated into two groups according to their preoperative BMI (40–45 and 45–50 kg/m^2^). All patients were supposed to undergo MGB/OAGB with an intestine length bypass of 180 cm [14]. 25 patients were put in group 1 (40–45 kg/m^2^), and 25 patients were put in group 2 (45–50 kg/m^2^).

Essential demographic information including gender, height, first BMI, and laboratory results including fasting blood sugar (FBS), triglycerides (TG), total cholesterol (TC), high-density lipoprotein cholesterol (HDL), low-density lipoprotein cholesterol (LDL), serum iron (Fe), ferritin, total iron-binding capacity (TIBC), 25-OH vitamin D, vitamin B12, international normalized ratio (INR), albumin, aspartate aminotransferase (AST), and alanine aminotransferase (ALT) were obtained preoperatively. Weight loss, BMI, and abovementioned laboratory data were reassessed in 15, 30, 90, and 180 days postoperatively.

All data were analyzed using IBM SPSS version 23.0 (Chicago, United States). Qualitative variables are expressed as number of patients and percentages. Quantitative variables are expressed as mean and standard deviation (SD). The Kolmogorov–Smirnov test was used to check the normal distribution of the variables. If they were normally distributed, the *t*-test and chi-square were used and if not, the Mann–Whitney and Wilcoxon tests were used. ANOVA and ANOVA with repeated measures were used to compare the results of each variable between more than two groups. *P* value <0.05 was considered significant.

### 2.1. Surgical Protocol

Under general anesthesia, the patient was put in the supine leg splitting position. A nasogastric tube and Foley catheter were inserted. A 150-millimeter (mm) Veress needle was placed in the left subcostal location, and pneumoperitoneum was established using carbon dioxide (CO_2_) to a maximum pressure of 15 millimeter mercury (mmHg). An 11-mm optical viewing trocar was inserted 15–20 cm below the xiphoid process to the left of the midline. The other laparoscopic ports were inserted under direct vision. The 12-mm right-handed working port was placed in the left midclavicular line, while the 5-mm left-handed working port was placed in the right midclavicular line. One 5-mm port was placed in the epigastrium for retracting the left lobe of the liver. Another 5-mm port was inserted in the left anterior axillary line at the level of the camera port. A narrow and long, approximately, 30 to 40 milliliter (ml) gastric pouch was created just below the crow's foot over a 32 French orogastric tube by four or five 60-mm Endo-GIA Tristaple purple loads (Covidien®/Medtronic). The gastrojejunostomy anastomosis was created 180 cm from the Treitz ligament with one 45-mm Endo-GIA Tristaple purple load (Covidien®/Medtronic) and was sutured with 2/0 Prolene twice to make sure there will not be any leak from the anastomosis site. After the surgery, all of the patients were sent to the recovery room and when they become conscious, they were sent to the surgery ward. All of the patients were discharged after 2 days of hospitalization if they did not face any early postoperative complications (e.g., peritonitis, leak from the anastomosis, bleeding, and surgical site infection.). We put all of our patients on full supplementation protocol as follows: first month, daily 10 ml of Sanostol® multivitamin syrup, and after that, until one year after the surgery, we recommended daily Pharmaton® multivitamin capsules.

## 3. Results

Our patients' mean age was 45 ± 8.36 years (range: 36–55) in group 1 and 46 ± 5.49 years in group 2 (range: 35–58) (*P*=0.15). 24% and 40% of groups 1 and 2 were male, respectively (*P*=0.22). Mean height of group 1 was 165.44 ± 4.98 cm (range: 150–175) and for the other group was 165.64 ± 8.45 cm (range: 150–175) (*P*=0.58).  Table 1 presents patients' body weight, BMI, and excess weight loss (EWL) in different phases following MGB/OAGB. Based on Table 1, all mentioned variables changed significantly in the two groups. Patients in groups 1 and 2 achieved %EWL of 60.51% and 46.64% after 6 months, respectively.

Table 2 presents laboratory factors and their changes in 3-month and 6-month periods after the surgery. As it can be seen that FBS, TG, TC, LDL, AST, and ALT have a down slope trend, which statistics showed significant changes in some of them; however, other factors like iron, ferritin, vitamin D, and vitamin B12 had been decreased at first and increased afterwards and none of them were significant statistically. None of our patients faced early postoperative complications. During the 6-month follow-up, none of our patients needed hospital admission for late complications or any reoperations. All of the data will be available for secondary analysis in necessary cases from the corresponding author through email address.

## 4. Discussion

Bariatric surgery as the absolute method of achieving weight loss in those with severe obesity is the favorable procedure used by surgeons. In this matter, a variety of techniques and their debatable concepts have been reviewed and studied to find the best technique of surgery to obtain the best outcomes and the least adverse effects [15].

The other issue about bariatric surgery is the intestinal bypassed length that causes fewer complications such as malabsorption and also acceptable weight loss. Studies have recommended different formulas for that [12–16].

In the current study, we aimed to compare whether more obese patients need longer intestinal bypass length or 180 cm which is a widely used and confirmed intestinal bypass length that works for them too according to their nutritional, metabolic, and weight loss changes following MGB/OAGB. Thus, two comparable groups with a BMI of 40–45 and 45–50 kg/m^2^ each containing 25 cases were compared from baseline.

Based on our findings, both groups lost their weight, BMI, and excess weight significantly. According to graphs' slope of these weight-associated variables, both groups had the same slope angle although group 2 had a higher starting point, but as our results showed, both groups had successful and progressive trend in their weight and BMI loss. As it can be seen in Table 1, after 6 months, both groups achieved BMI <35 kg/m^2^. However, %EWL was higher in patients with lower preoperative BMI level and it can be explained through the EWL formula and its instinct, and patients with higher BMI had more weight to lose in a specific amount of time; therefore, using this indicator is not representative of all changes in weight correctly and completely. Group 1 patients achieved a mean %EWL of 60.51% 6 months after the surgery; however, group 2, which had higher BMI and higher excess weight, achieved losing 46.64% of their weight during this time by the 180 cm intestinal anastomosis.

In a study conducted by Mahawar et al., they presented that a bypass length of 100–200 cm has acceptable outcomes and few possible complications such as malabsorption [16], while in a study conducted by Carbajo et al., they assessed patients who had undergone MGB/OAGB with 250–300 cm bypass length and found malabsorption in 1.1% of them that was compatible with prevalence of protein malabsorption presented in other studies [14]. In a study conducted by Lee et al., they compared outcomes of MBG/OAGB surgery in which bypassed length was chosen based on patients' BMI. In their study, bypassed limb was 150 cm, 250 cm, and 350 cm for those with a BMI of less than 40, 40–50, and over 50 kg/m^2^, respectively. BMI reduction in their patients were 10.7, 15.5, and 23.3, respectively. In general, they concluded that decision on length of the limb should be made based on BMI. In addition, they presented that for those with lower BMI, choosing the appropriate limb length is more serious [13].

Other variables including metabolic and nutritional factors were assessed among two groups as well with intervals of three and six months after the surgery. In general, based on Table 2, we found that metabolic factors tend to change in an acceptable manner, such as decrease of TG, TC, LDL, FBS, and liver enzymes. It is reasonable that patients with higher BMI have higher levels of TG, TC, LDL, and FBS at the baseline, but both groups had progressive declined slope. Bariatric surgeries reported controversial results on levels of liver enzymes, in which some of them mentioned these surgeries can deteriorate the liver function; however, others suggested that as the weight decreases, the grade of fatty liver decreases and they can cause improvement in the liver function tests. According to the baseline levels, other factors including HDL, serum iron, ferritin, vitamin D, and vitamin B12 were simultaneously decreased in 3 months and then increased after 6 months, and they could be due to postoperative supplement use. Those changes were not statistically significant between both groups. It should be noted that HDL, which is the antiatherosclerotic lipid, was decreased after the surgery and it is an unexplained, unusual, and unfavorable change, which needed further evaluations.

The study by Rutledge and Walsh presented that gastric ulcer and iron deficiency anemia were the most prevalent and significant postoperative complications in their patients [17]. In our study, serum iron, ferritin, and vitamin B12, which are important indicators of intestinal absorption, were changed in the same way all together after 3 and 6 months.

The other study conducted by Lee et al. assessed serum glucose changes among obese people with type-2 diabetes mellitus. They found that patients with lower BMI had significantly lower liver enzymes and C-peptide preoperatively. In addition, they found that patients lipid profile and FBS returned to normal ranges in approximately 90% of the patients, but these changes were not significantly different among those with a BMI of less than 35 or more than 35 kg/m^2^ [18].

In a study in 2015, sleeve gastrectomy was compared with MGB/OAGB regarding lipid profile. They presented that although in 3- and 6-month follow-up, lipid profile changes to normal ranges were higher in MBG/OAGB, but after one year, both techniques had similar outcomes [19]. Other studies in this regard have assessed other techniques of bariatric surgery except MGB/OAGB, but outcomes of all studies are unanimous about positive effects of bariatric surgery on the glycolipid profile. In addition, most of the studies are in favor of bypass techniques in comparison to techniques with restrictive approach (e.g., sleeve gastrectomy) [20–22].

To resolve our limitations, we suggest researchers to evaluate each single anastomosis length between two different groups of patients and different intestinal bypass length for patients with the same BMI to reach to a consensus for choosing appropriate intestinal bypass length for patients with different BMI category. Furthermore, larger sample size, randomized controlled trials, and longer postoperative follow-up can decrease the biases of choosing the patients and evaluating the results of bariatric surgery in the long term.

## 5. Conclusions

We found that 180-cm intestinal bypass length can make significant changes in weight and BMI in both groups of patients. Glycolipid changes were acceptable in both groups.

## Figures and Tables

**Table 1:** Weight, BMI, and excess weight loss changes trend following 6 -month follow-up after the laparoscopic one anastomosis gastric bypass.

Variables	BMI groups (kg/m^2^)	Baseline	15 days	30 days	3 months	6 months	*P*_1_ value	*P*_2_ value
Weight, kg (mean ± SD)	40–45 (*n* = 25)	113.80 ± 8.65	106.88 ± 9.02	101.72 ± 7.92	91.20 ± 8.20	81.04 ± 7.71	<0.001	0.001
	45–50 (*n* = 25)	132.56 ± 11.86	125.68 ± 10.11	120.64 ± 9.88	109.72 ± 11.43	95.96 ± 11.53	<0.001	
BMI, kg/m^2^ (mean ± SD)	40–45 (*n* = 25)	41.53 ± 1.44	37.50 ± 7.16	37.15 ± 1.64	33.25 ± 1.94	29.44 ± 2.42	<0.001	0.007
	45–50 (*n* = 25)	48.36 ± 2.28	45.88 ± 2.48	44.03 ± 2.26	39.96 ± 2.22	34.89 ± 1.82	<0.001	
Excess weight loss (%)	40–45 (*n* = 25)	—	14.79 ± 4.79	23.04 ± 5.73	44.24 ± 12.92	60.51 ± 24.26	<0.001	0.007
	45–50 (*n* = 25)	—	10.77 ± 4.11	15.45 ± 6.06	29.12 ± 15.22	46.64 ± 19.04	<0.001	

*P*_1_ value is the mean differences of each variable in each group data during the time and was calculated using ANOVA with repeated measures. *P*_2_ value is the comparison of both the groups for each variable in multiple measures and was calculated with ANOVA.

**Table 2:** Metabolic and nutritional state results of each BMI group from baseline to 6 months after the laparoscopic one anastomosis gastric bypass.

Variables (mean ± SD)	BMI groups (kg/m^2^)	Normal laboratory reference range	Baseline (*n* = 50)	3 months (*n* = 50)	6 months (*n* = 50)	*P*_1_ value	*P*_2_ value
TG (mg/dL)	40–45	35–200	127.72 ± 51.58	119.32 ± 39.13	103.92 ± 42.797	<0.001	0.02^*∗*^
	45–50		183.00 ± 71.44	162.80 ± 68.30	137.88 ± 65.63	<0.001	
TC (mg/dL)	40–45	Normal: <200 Borderline: 200–240 High risk: 240–400 Very high risk: >400	181.36 ± 37.50	160.24 ± 33.69	150.84 ± 22.99	<0.001	0.03^*∗*^
	45–50		209.08 ± 42.75	174.04 ± 21.13	156.60 ± 21.90	<0.001	
HDL (mg/dL)	40–45	Men: >35 Women: >45 Desirable value: 40–60 Protect against CHD: >60 High risk for CHD: <20	47.32 ± 9.77	42.80 ± 5.17	44.40 ± 7.46	0.92	0.14
	45–50		54.08 ± 14.32	49.16 ± 10.52	45.52 ± 6.13	0.73	
LDL (mg/dL)	40–45	Desirable: <130 Borderline: 130–160 High risk: >160	108.28 ± 32.46	99.32 ± 21.39	92.52 ± 16.87	0.44	0.59
	45–50		117.20 ± 25.46	109.64 ± 14.69	97.60 ± 14.50	0.29	
Fe (mcg/dL)	40–45	60–150	87.88 ± 8.85	77.48 ± 21.75	81.64 ± 30.13	0.95	0.40
	45–50		92.08 ± 15.34	81.36 ± 12.41	89.08 ± 18.09	0.12	
Ferritin (ng/mL)	40–45	20–200	65.20 ± 15.97	57.64 ± 26.66	59.20 ± 17.84	0.70	0.13
	45–50		65.08 ± 20.88	62.18 ± 23.29	64.68 ± 48.86	0.47	
TIBC (mcg/dL)	40–45	240–450	257.76 ± 52.26	292.2 ± 47.57	304.56 ± 52.39	0.15	0.054
	45–50		280.88 ± 75.94	285.92 ± 65.66	298.40 ± 75.31	0.55	
Vit B12 (pg/mL)	40–45	200–900	349.64 ± 112.06	342.68 ± 126.76	354.52 ± 107.18	0.44	0.03^*∗*^
	45–50		300.56 ± 63.82	310.52 ± 71.25	308.60 ± 63.68	0.53	
Vit D (ng/mL)	40–45	Severe deficient: <10 Deficient: 10–30 Optimal level: 31–80 High normal: 91–99 Overdose: 100–150 Toxic: >150	40.20 ± 4.93	36.77 ± 5.89	37.80 ± 6.30	0.57	0.99
	45–50		38.13 ± 3.88	35.82 ± 6.50	35.78 ± 9.86	0.64	
FBS (mg/dL)	40–45	Normal: <100 Prediabetes: 100–125 Diabetes: >125	96.60 ± 10.42	85.72 ± 8.44	82.12 ± 6.61	0.02^*∗*^	0.21
	45–50		103.72 ± 15.65	93.56 ± 11.41	84.76 ± 7.94	0.03^*∗*^	
INR	40–45	<1.5	1.08 ± 0.11	1.10 ± 0.07	1.11 ± 0.06	0.43	0.53
	45–50		1.14 ± 0.14	1.12 ± 0.11	1.14 ± 0.09	0.33	
Alb (g/dL)	40–45	3.5–5.5	4.47 ± 0.24	4.35 ± 0.31	4.29 ± 0.28	0.94	0.69
	45–50		4.54 ± 0.20	4.40 ± 0.19	4.31 ± 0.19	0.72	
AST (IU/L)	40–45	<40	21.48 ± 5.35	18.28 ± 3.31	16.84 ± 3.14	0.60	0.61
	45–50		23.16 ± 5.24	19.64 ± 2.97	18.16 ± 3.68	0.57	
ALT (IU/L)	40–45	Male: <40 Female: <30	25.60 ± 6.82	20.00 ± 4.97	16.92 ± 3.66	0.43	0.39
	45–50		25.56 ± 8.64	20.88 ± 5.34	18.72 ± 4.05	0.38	
ALP (IU/L)	40–45	Female: 64–306 Male: 80–306	198.28 ± 18.32	192.20 ± 17.40	194.64 ± 17.92	0.29	0.47
	45–50		187.20 ± 27.43	184.04 ± 26.81	195.24 ± 19.53	0.66	

TG, triglyceride; HDL, high-density lipoprotein; CHD, coronary high disease; LDL, low-density lipoprotein; TC, total cholesterol, Fe, iron; TIBC, total iron-binding capacity; Vit, vitamin; FBS, fasting blood glucose; INR, international normalized ratio; Alb, albumin; AST, aspartate aminotransferase; ALT, alanine aminotransferase; ALP, alkaline phosphatase; g: gram; mg: milligrams; ng: nanograms; mcg: micrograms; pg: picograms; L: liter; dL: deciliter; mL: milliliter; IU: international unit. *P*_1_ value is the mean differences of each variable in each group data during the time and was calculated using ANOVA with repeated measures. *P*_2_ value is the comparison of both the groups for each variable in multiple measures and was calculated with ANOVA. ^*∗*^Indicates significant *P* value.

## Data Availability

All of the data will be available for secondary analysis in necessary cases from the corresponding author upon request through email.
